# Observation of the extent of smoking in a mental health inpatient facility with a smoke-free policy

**DOI:** 10.1186/1471-244X-14-94

**Published:** 2014-03-29

**Authors:** Paula Wye, Leanne Beth Gow, Jude Constable, Jenny Bowman, Sharon Lawn, John Wiggers

**Affiliations:** 1 University of Newcastle, University Drive, Callaghan, New South Wales (NSW) 2308, Australia; 2 Hunter Medical Research Institute (HMRI), Lot 1 Kookaburra Circuit, New Lambton Heights, NSW 2305, Australia; 3 University of Newcastle Priority Research Centre for Health Behaviour (PRCHB), Room 271, Level 2, David Maddison Building, Cnr King and Watt Streets, Newcastle, NSW 2300, Australia; 4 Hunter New England Population Health (HNEPH), Longworth Ave, Wallsend, NSW 2287, Australia; 5 Hunter New England Mental Health Service, Mater Hospital, Cnr Edith and Platt Streets, Waratah, NSW 2298, Australia; 6 Department of Psychiatry, Flinders University, PO Box 2100, Adelaide, South Australia 5001, Australia

**Keywords:** Nicotine dependence treatment, Smoke-free policy, Mental health, Smoking

## Abstract

**Background:**

People with a mental illness experience a higher burden of smoking-related disease. Smoke-free policies in mental health facilities provide an opportunity to reduce smoking-related harms for patients and staff alike. Limited evidence regarding the effect of such policies on preventing smoking in mental health facilities has been reported. The aims of this study are to describe the extent of smoking and the provision of nicotine replacement therapy (NRT) to patients in a mental health facility with a smoke-free policy.

**Methods:**

Cross-sectional studies of smoking (cigarette butt count and observed smoking) and nicotine dependence treatment (patient record audit) were undertaken over 9 consecutive weekdays in one mental health facility in Australia. A smoke-free policy incorporating a total smoking ban and guidelines for treating nicotine dependence among patients was implemented in the facility 4 years prior to the study.

**Results:**

Two thousand one hundred and thirty seven cigarette butts were collected and 152 occasions of people smoking were observed. Staff members were observed to enforce the policy on 66% of occasions. Use of NRT was recorded for 53% of patients who were smokers.

**Conclusion:**

Implementation of the smoke-free policy was less than optimal and as a consequence ineffective in eliminating smoking and in optimising the provision of NRT. Additional strategies to improve the provision of nicotine dependence treatment to patients and the monitoring of adherence are needed to ensure the intended benefits of smoke-free policies are realised.

## Background

People with a mental illness experience a higher burden of smoking-related disease, a key factor in their having a markedly shorter life expectancy [1,2]. This inequitable disease burden is in part a function of a greater prevalence of smoking, one that is at least double that of the general population [3,4]. Smoking bans in a variety of settings have been effective in reducing the prevalence of smoking and exposure to second-hand smoke [5]. Similarly, the use of nicotine replacement and other pharmacotherapies are effective in aiding the management of nicotine withdrawal and in aiding smoking cessation [6]. Given such evidence, clinical guidelines recommend the implementation of smoke-free policies in health facilities that incorporate both a ban on smoking and the provision of nicotine dependence treatment to patients. The provision of nicotine dependence treatment is of particular importance as a means of treating the negative effects of nicotine withdrawal among patients, and of limiting the likelihood of patient discharge against medical advice and the taking of leave to smoke [7,8]. The implementation of such policies in mental health facilities has particular salience as a means of addressing the tobacco-related iatrogenic effects of admission [9], effects that include an increased risk of patient smoking initiation [10]; smoking relapse [11]; and heavier smoking [9,12].

Inconsistencies in the implementation of smoke-free policies in mental health facilities have been reported in a number of countries [8,13,14]. Past evaluation of the impact of such policies has primarily involved measurement of staff perceptions regarding the enforcement of such policies and the impact of such policies on the provision of psychiatric care, rather than on either the elimination of smoking within facilities, or the provision of nicotine replacement therapy (NRT) [5,13,15-17]. Of the limited number of studies that have directly measured impact on the prevalence of smoking, continued smoking within facilities has been a commonly reported finding [13,17].

In past studies of the extent of smoking within facilities with a smoke-free policy, the measurement of smoking has primarily involved patient and/or staff report [10,12,13]. As a consequence, given the social desirability limitations of self-report measurement generally [18] and of such measurement for patients in the context of a smoke-free health facility in particular, understanding of the extent of smoking within mental health facilities with a smoke-free policy is constrained. Given this, and given the suggestion that the intended health, clinical and safety benefits of smoke-free policies in mental health facilities are not being fully realised, an observational study was undertaken to describe the extent of smoking and the provision of NRT to patients in a mental health facility with a smoke-free policy.

## Methods

### Setting

A cross-sectional study was conducted in a single mental health inpatient facility in the state of New South Wales, Australia. The research was carried out in compliance with the Helsinki Declaration, and approved by the Hunter New England Human Research Ethics Committee (Approval No. 10/10/20/5.05). The study was conducted over nine consecutive weekdays in July/August, 2010.

The facility contained six separate locked units (patients unable to move beyond the unit without permission) with 96 beds in total and an annual bed occupancy rate of 95%. All indoor areas of the facility were fitted with smoke detectors. Voluntary patients, approximately 70% of admissions, were able to obtain unsupervised leave passes from the facility. Visiting hours for the facility were from mid-afternoon to early evening.

A smoke-free policy was introduced in the facility in 2006 that incorporated a ban on smoking in all indoor and outdoor areas by all persons (staff, patients, visitors). Visitors were informed verbally, through written visitor information and signage that smoking was not permitted and if they were identified to be smoking they would be asked to leave. The policy also required the provision of nicotine dependence treatment to all dependent smokers.

Specifically, the policy required all patients, on admission to the facility to: relinquish all tobacco products; have their smoking status recorded; and if they were a smoker, to have their nicotine dependence status recorded and be provided brief cessation advice and NRT in the form of transdermal patches and additional intermittent nicotine replacement therapy in the form of gum, lozenge and inhaler if needed. The policy required the recording of the provision of such NRT. Other treatment modalities such as varenicline, bupropion or specialist behaviour support were not available. NRT was made available from nurse unit stations and during routine medication dispensing activities and recorded when provided.

Strategies implemented to increase adherence to the smoke free policy involved: changes to local treatment policies and guidelines; training for all clinical and medical staff; routine site visits to support nicotine dependence treatment; and audit and feedback of NRT provision based on medical file audits. The training and feedback regarding NRT provision was provided both formally through in-service and staff meetings, and informally on a one-on-one basis to clinical staff by an implementation officer.

### Sample

#### ***Facility units and external courtyards***

Previous studies have reported high levels of adherence with smoke-free policies within buildings and enclosed areas [19], but not in unenclosed outdoor areas [20]. Such outdoor areas in inpatient mental health facilities have been suggested to be a focus for smoking [10]. Given this, and given that smoking was reported to be negligible inside the study facility buildings, but common in the attached outdoor courtyards, the study was conducted in such courtyards.

Five (92 beds) of the six units within the study facility were eligible for inclusion in the study as each had an attached external courtyard: psychiatric intensive care unit (8 beds); older person’s unit (18 beds); dual diagnosis unit with two sections, each with a courtyard (12 beds and 10 beds); and two general acute units (22 beds each). One of the general acute units had two courtyards in use, providing a total of seven courtyards as the focus of the study (Table 1).

**Table 1:** Cigarette butts collected, and number of occasions of people smoking in each unit courtyard

**Courtyard location**	**Cigarette butts counted (n, % of total)**		**Occasions of people observed to smoke (n, % of total)**		**Occasions of people who did not smoke (n, % of total)**	
Intensive care unit	0		1	.5%	20	10%
Older person’s unit	0		0		3	1.5%
General acute 1	423	20%	57	38%	54	27%
General acute 2						
East courtyard	466	22%	32	21%	52	26%
South courtyard	72	3%	2	1%	18	9%
Dual diagnosis						
North courtyard	394	18%	23	15%	31	16%
South courtyard	782	37%	37	24%	20	10%
Total	2137		152		198	

Each eligible unit and attached courtyard was wholly located within the building, with the courtyard only able to be accessed through the unit itself. The courtyards were able to be accessed from approximately 7 am to 10 pm 7 days a week. The size of the courtyards ranged from 92 m^2^ to 152 m^2^. All of the courtyards were unroofed.

All occasions of persons (patients, staff, and visitors) who entered the external courtyards constituted the study sample.

#### ***Prevalence of recorded nicotine dependence treatment for patients***

The recorded provision of nicotine dependence treatment was assessed for all patients who were smokers and admitted during the nine consecutive week-day study observation period, and for smokers admitted for an equivalent period one month before and one month after this period.

### Data collection procedures

Facility staff and patients were informed of the study data collection procedures one week before study commencement through normal communication channels including management meetings, staff meetings, in-service training sessions and a broadcast management email to all staff, and through routine clinical meetings with patients. The information provided to staff and patients advised that the study was being conducted to determine adherence with the smoke-free policy.

#### ***Extent of smoking***

Observational data regarding the extent of smoking was collected in two ways:

1) Occasions of observed smoking

  Direct observation of all occasions of people (patients, staff, visitors) smoking in the courtyards was conducted over nine consecutive weekdays in July/August (winter). Each courtyard was observed for 25 minutes on two occasions each weekday, once in the morning between 7 am and 10.25 am, and once in the afternoon between 3 pm and 6.25 pm to avoid conflict with patient activities conducted during the middle of the day.

  A random sequence generator was used to assign the observation times to each courtyard each day. Over the 9 days of observation, each courtyard was observed on 18 occasions, totalling 126 observation periods involving 52 hours and 50 minutes of observation across all courtyards.

  A pen and paper observational recording tool (20 items) was developed based on that reported by Lawn [11]. Pilot studies of the data collection tool and procedures were conducted to refine the tool.

  The observation tool was completed on a real time basis by one trained study observer who was not a member of staff (BG) seated in the courtyard in a visible non-obtrusive position with line-of-sight to all parts of the courtyard. To determine the reliability of recorded observations of smoking, an inter-rater reliability assessment was conducted in 2 of the units during an earlier pilot study in which the first author (PW) was the gold standard observer for comparison with the study observer (BG). One hundred per-cent agreement was obtained between the observers for all items on the observation recording tool.

2) Cigarette butt count

  At the conclusion of each observation period the study observer collected, counted and removed all cigarette butts from the ground, ash trays, planter boxes, bins, and gardens within each courtyard.

#### ***Courtyard environment***

As weather conditions have been reported to impact on the extent of outdoor smoking in hospitals [21], the weather conditions for each observation period were recorded via the observation tool, as was the presence of smoke free signage during the initial observation period in each courtyard.

#### ***Prevalence of recorded nicotine dependence treatment***

All medical records of admitted patients during three periods were retrospectively audited post discharge to determine the extent to which nicotine dependence treatment was recorded as being used by patients.

The medical record audit involved a review of six locations within the medical record where nicotine dependence treatment could be recorded: admission forms; review forms; clinical notes; medication chart; electronic discharge summary; and diagnoses summary [22]. The audit was conducted by a clinician experienced and trained in the conduct of medical record audits. An inter-rater reliability analysis of the audit methodology has previously been reported [23].

#### ***Patient demographic, smoking and facility characteristics***

Data regarding patient demographics, smoking status and facility characteristics were obtained via the medical record audit. Unit managers were asked to indicate the number of visitors to the unit each day. Number and type of staff on duty during each day of the study was obtained from the Unit Managers of each facility.

### Measures

#### ***Occasions of people using the courtyards***

The observer recorded for each occasion a person who entered the courtyard, whether the person was a member of staff based on their wearing of a mandatory staff identification badge. All other persons entering the courtyard were recorded as patient/visitor, as no observational basis for distinguishing between such persons was available. A person was counted more than once if they re-entered the courtyard during a single observation period.

#### ***Extent of smoking***

1) Occasions of observed smoking

  For every occasion a person entered the courtyard, the study observer recorded whether they smoked whilst in the courtyard (lighting up, stubbing out, smoking; not smoking). When a staff member was identified as being in the courtyard at the same time as a person who was smoking, the observer recorded whether the staff member enforced the smoke-free policy (observed to speak to smoker who subsequently stopped smoking).

2) Cigarette butt count

  All cigarette butts were removed from all courtyards on the evening prior to the commencement of the study. Thereafter, the number of cigarette butts collected and removed from each courtyard was recorded at the conclusion of each observation period. This included all butts that accumulated over the middle weekend of the study period being collected at the conclusion of the first observation on the following Monday morning.

#### ***Courtyard environmen***

The weather conditions for each observation period were recorded as: sunny, cloudy or raining, and temperature was classified as <10°C; 10-20°C, >20°C based on weather reports for the day. Presence of smoke free signage was recorded as either yes/no.

#### ***Prevalence of recorded nicotine dependence treatment***

The recorded provision of the following elements of nicotine dependence treatment was noted for each patient (yes/not recorded): smoking status; provision of brief advice to quit; and use of any NRT [22].

#### ***Patient demographic, smoking and facility characteristics***

Information regarding whether a patient was identified as a smoker through the assessment process (yes, no), the treating unit at discharge, and admission and discharge dates for each patient were obtained from the medical record audit. Unit managers were asked to indicate the number of visitors to the unit.

### Analysis

#### ***Patient demographic, smoking and facility characteristics***

Descriptive statistics were used to describe the number and characteristics of patients and of the facilities, and the average number of visitors.

#### ***Occasions of observed smoking and cigarette butt count***

Descriptive statistics were used to describe the number and characteristics of all people observed to enter the courtyards, whether they were observed to smoke and the number of cigarette butts collected.

#### ***Prevalence of recorded nicotine dependence treatment***

Descriptive statistics were used to describe recorded patient smoking status and provision of nicotine dependence treatment. Admission and discharge dates of patients were used to calculate the number of patients in each unit during the study period.

All data analyses were conducted using SPSS (Version 19) [24].

## Results

### Sample

A total of 165 people were patients of the five units for at least one day of the study period. Unit managers indicated an average of five people visited the study units each day. Approximately 80 individual staff members were on duty on any given morning or afternoon shift throughout this period, the majority of whom were nurses (60%).

#### ***Extent of smoking***

• Courtyard and weather characteristics

  Forty-five per cent of observations were conducted when the weather was rainy, and 65% occurred when the outdoor temperature was less than 10°C. No observations were recorded where the temperature exceeded 20°C. Smoke-free signage was observed in all courtyards except the intensive care unit courtyard.

• Occasions of observed smoking

  In total, 350 people were observed in the courtyards over the 9 days of observation, 3% (n = 12) of whom were staff and 97% patients/visitors (n = 338). One hundred and fifty two occasions of people smoking were observed (43% of all occasions observed) (Table 1). The prevalence of smoking occurred equally between the morning (49%) and the afternoon (51%) observation periods. No members of staff were observed to smoke. Almost all occasions of people smoking (99%) occurred in four of the seven study courtyards connected to two units (general acute and both dual diagnosis courtyards).

  In 6 (5%) observation periods, staff (n = 12) were present in the courtyards at a time when another person was observed to be smoking. On these occasions 8 staff members (66%) were observed to enforce the non-smoking policy. No staff members were observed to provide NRT in the courtyard.

• Cigarette butt count

  In total, 2137 cigarette butts were collected (Table 1). All of the butts were collected from 4 of the 7 courtyards. Fifty-five per cent of butts (1176) were collected from the two dual diagnosis courtyards, and 45% (961) were collected from the two general acute unit courtyards.

### Prevalence of recorded nicotine dependence treatment

Of the 165 admitted patients to the units during the observation period, 76 (46%) were identified in the medical record as smokers.

The proportion of patients in each unit who were recorded as smokers varied from 6% (n = 2) in the older persons unit, 40% (n = 16) and 46% (n = 19) in the two general acute units, 66% (n = 6) in the intensive care unit, and 89% (n = 33) in the dual diagnosis unit.

Twenty-one of the smokers (28%) were recorded to have received advice to quit, and 40 (53%) were recorded to have used some form of NRT during their admission.

The prevalence of patients who were smokers, and the proportion of smokers recorded to have used any NRT during the observation study period were similar to the equivalent periods one month prior to and the month following (Table 2).

**Table 2:** Recorded provision of nicotine dependence treatment prior to, during and following the observation period

**Audit period (patients discharged)**	**Smokers**		**Quit advice**		**Used any NRT**	
	**N**	**%**	**N**	**%**	**N**	**%**
1 month prior to observation period (N = 143)	65	45	19	29	37	57
Observation period (N = 165)	76	46	21	28	40	53
1 month after observation period (N = 148)	68	46	13	19	38	56

## Discussion

The study found that despite the implementation of a smoke-free policy incorporating a total smoking ban and the provision of NRT four years previously, a substantial amount of smoking continued in the inpatient facility. Over the consecutive nine week day observation period 2137 cigarette butts were removed from the courtyards and 152 occasions of people smoking were observed. Few members of staff were observed in the courtyards, and when present, were observed to enforce the smoke free policy on 66% of occasions. Such findings suggest a less than optimal implementation of the smoke-free policy. As a consequence the findings suggest the courtyards function as default smoking areas, subverting the intention of a smoke-free policy.

The finding of ongoing smoking within the facility despite a smoke-free policy suggests that the previously reported inconsistent implementation of such policies in mental inpatient facilities in Australia is ongoing [10,14,25]. Such a conclusion is consistent with similar findings in the United Kingdom [17]. Ratschen et al. reported that 81% of staff from UK healthcare trusts with mental health inpatient facilities and total smoking bans reported ongoing patient smoking within such facilities, with more than one third reporting that smoking occurred daily [17]. Such findings suggest that additional strategies are required to enhance the effectiveness of smoke-free policies if the tobacco-related iatrogenic effects of admission to mental health facilities are to be prevented, and the intended benefits of a smoke-free policy are to be realised.

A marked variability in the extent of observed smoking was evident, with all cigarette butts and 99% of the observed occasions of people smoking being observed in just four of the seven court yards. Such a finding may be attributable to the difference between the units in the prevalence of smokers or of smokers who are nicotine dependent as indicated in this study and others [9,26]. In addition, unit differences in other patient clinical characteristics, staffing ratios, level of policy adherence monitoring and clinical management of smoking may have also contributed to the variable findings between units. Given such variability, the potential exists for reducing the prevalence of smoking in the facility by identifying the clinical and management practices of those units associated with lower levels of observed smoking, and applying them, where applicable, to the units with observed higher levels of smoking.

The levels of observed smoking are consistent with studies that have suggested that unenclosed outdoor areas present particular challenges in terms of achieving smoke free policy adherence [20]. One approach to enhancing the effectiveness of smoke-free policies in such areas involves consistent enforcement of adherence [27]. In this study, in the majority of occasions (66%) when a staff member was present in the courtyard when a person was observed to be smoking, the staff member was observed to enforce the policy. As no previous study has quantified the observed level of staff enforcement in occasions of people smoking, the extent to which this finding is consistent with levels of enforcement reported elsewhere is unknown. As a number of studies have suggested that staff report a lack of skill and confidence in enforcing a smoke-free policy, or do not support such a policy, the observed non-enforcement in 34% of occasions of smoking may have been attributable to such reasons [14,16]. Further research exploring barriers in specific occasions of non-enforcement, and assessing the effectiveness of training in addressing such barriers would be of benefit [14,16].

In addition to the observed level of non-enforcement, the continued smoking in the courtyards, as indicated by the number of cigarette butts collected, is likely to be, in part, a function of the limited presence of staff in the courtyards. In only 5% of observation periods was a staff member observed to be present in a courtyard whilst a person was observed to be smoking. The possibility exists that due to resource constraints, staff may not have had sufficient time to be present in the courtyards to monitor people smoking [16]. Similarly, members of staff may have chosen to not enter the courtyard as a consequence of their lack of confidence and perceived skill in enforcement [14,16]. Staff may also have chosen not enter the courtyards to avoid exposure to tobacco smoke. Regardless of the explanation for the limited staff presence in the courtyards, the findings suggest a need to enhance the monitoring of smoking in a smoke free facility [28]. The systematic provision of monitoring and feedback regarding particular aspects of clinical care has been demonstrated to be an effective strategy in facilitating clinical practice change [29]. Given the likely inability of staff to be constantly present in external areas of mental health facilities, implementation of a systematic monitoring and feedback mechanism, with the observational approach applied in this study representing one such mechanism, may represent one means of achieving this.

The provision of NRT has been reported to support the implementation of smoke-free policies through its contribution to managing nicotine withdrawal [7,8]. The medical record audit reported in this study found that NRT was provided to approximately half of the patients recorded to be smokers, a level of care that was consistent with that in the month prior to and the month following the observation period, suggesting limited reactivity to the study design. The level of such care provision (53%) was higher than that previously reported in a study using the same measurement methods conducted in the same study facility four years previously prior to the introduction of the smoke-free policy (0%). Such a finding suggests an increase in nicotine dependence treatment had occurred in the period following the introduction of the smoke-free policy [23]. An increase in nicotine dependence treatment provision has similarly been reported in a number of other studies following the implementation of smoke-free policies [13].

Despite the suggested improvement in the provision of NRT, the level of such care found in this study was less than optimal for minimising the negative effects of nicotine withdrawal [8]. Such a finding is consistent with previous studies of nicotine dependence treatment in mental health facilities generally [10,23] and in facilities with smoke-free policies in particular [17,30], suggesting that the potential exists for the effectiveness of such policies to be enhanced by the improved provision of NRT to smokers. Clinical guidelines recommend that a systems approach involving multiple practice change strategies such as clinical decision aids and prompts, education and training of staff, and audit and feedback of treatment provision is required to improve clinician provision of such care [29]. The findings of this study suggest further investment in such an approach is required if the benefits of NRT as a means of managing nicotine withdrawal are to be optimised.

A number of the characteristics of this study need to be considered when interpreting its findings. First, the number of occasions of people observed to smoke is likely to have been an underestimate of the normal extent of smoking within the facility as staff and patients were forewarned of the study, and the observer was clearly visible. Similarly, the potential exists for smokers to have avoided smoking for the brief period the observer was present in the courtyard. The observed extent of staff enforcement of the policy may also have been an over-estimate of that which normally occurs given the presence of the observer. In the event of such effects occurring for patients and staff, the principal findings of the study regarding inadequate implementation of the smoke free policy are strengthened.

Second, the number of cigarette butts collected may have been an underestimate of the number of cigarettes smoked in the courtyard as cigarette butts may have been disposed of by means other than in the waste containers in the courtyards (for example under planters, or in toilets), and patients may have collected the discarded cigarette butts of other patients. The findings may also reflect a lower than normal level of smoking due to the cold and rainy weather, a factor previously reported to reduce the extent of patient smoking [21].

Third, inability of the study to distinguish between patients and visitors limited its ability to accurately quantify the extent of smoking by patients, and the contribution of visitors to the extent of observed smoking. All instances of observed smoking in the morning observation periods involved smoking by patients as visiting hours were in the afternoon. Based on an average of 5 visitors per day visiting the units, and no observed difference in the number of observed smokers between the morning and afternoon periods, it is considered that the large majority of observed smoking was by patients.

Fourth, as the study was conducted in a single facility in one health district it is not known if the findings can be generalised to other mental health hospital settings within the state, country or to other countries. However, given previously reported findings of an inconsistent implementation of smoke-free policies in mental health facilities across the state [10], and in a number of countries, a similarly limited effectiveness of such policies in eliminating smoking could be expected, given similar facility characteristics [13,17]. Further studies are required to determine the extent to which the findings are generalizable to other jurisdictions.

Fifth, the accuracy of the recording of smoking and nicotine dependence treatment in the medical record is unknown, as is the appropriateness of the dosage, type or quality of use of such treatment. As a consequence, the adequacy of the nicotine dependence treatment in terms of successfully managing patient nicotine withdrawal is unknown. Whilst the provision of such treatment has the potential to mitigate withdrawal effects, the achievement of such an outcome is dependent on an appropriate dosage and consistent usage. As no previous studies have reported the quality of provision or use of such treatment in the context of a smoke-free policy in a mental health facility, further studies of such treatment and its use are required [2,6-8,14,22,31].

Finally, the aggregate measures of the extent of smoking used in this study are likely to have masked a varied response by individual patients to the smoke-free policy. It is possible that some patients stopped smoking completely, whilst others may have smoked a greater number of cigarettes [12], and some may have taken leave and smoked offsite. Further research is required to obtain a better understanding of the variability of individual patient response to a smoke-free policy as a means of developing more individually tailored strategies for managing patient nicotine withdrawal and hence facilitating greater adherence to such a policy.

## Conclusion

Despite the introduction of a comprehensive smoke-free policy incorporating nicotine dependence treatment guidelines, substantial smoking was evident within the mental health facility. Only half of the patients used nicotine dependence treatment. More consistent and adequate implementation of such treatment, coupled with appropriate monitoring and response to observed smoking is required to enhance the effectiveness and benefits of smoke-free policies.

## Abbreviations

NRT: Nicotine replacement therapy.

## Competing interests

The authors declare that they have no competing interests.

## Authors’ contributions

PW conceived the study, made substantial contribution to the design of the study, the analysis and interpretation of data, drafted the manuscript, and has read and approved the final manuscript. LBG contributed to the study design, undertook the observations and collected the data, assisted with drafting the manuscript, and has read and approved the final manuscript. JC contributed to the conception and coordination of the study, participated in drafting the manuscript, and has read and approved the final manuscript. JB contributed to the analysis and interpretation of data, was involved in critical revision for important intellectual content, and has read and approved the final manuscript. SL contributed to the study design, has been involved in drafting the manuscript, and has read and approved the final manuscript. JW contributed to the conception and design of the study, interpretation of data, has critically revised drafts for important intellectual content, and has read and approved the final manuscript.

## Pre-publication history

The pre-publication history for this paper can be accessed here:

http://www.biomedcentral.com/1471-244X/14/94/prepub
